# Omnidirectional Triboelectric Nanogenerator Operated by Weak Wind towards a Self-Powered Anemoscope

**DOI:** 10.3390/mi11040414

**Published:** 2020-04-14

**Authors:** Nay Yee Win Zaw, Hyeonhee Roh, Inkyum Kim, Tae Sik Goh, Daewon Kim

**Affiliations:** 1 Department of Electronic Engineering, Institute for Wearable Convergence Electronics, Kyung Hee University, Yongin 17104, Korea; 2 Department of Orthopaedic Surgery and Biomedical Research Institute, Pusan National University Hospital, Busan 49241, Korea

**Keywords:** wind, triboelectric nanogenerator, directionality, polytetrafluoroethylene, sensor, strip

## Abstract

Wind is a great sustainable energy source for harvesting due to its abundant characteristic. Typically, large space, loud noise, and heavy equipment are essential for a general wind power plant and it is solely operated by big-scale wind. However, wind energy can be efficiently harvested by utilizing the triboelectric nanogenerator due to its abundance, ubiquity, and environmentally friendliness. Furthermore, a few previously reported wind-driven triboelectric nanogenerators, which have the bulk fluttering layer by wind, still show difficulty in generating electricity under the conditions of weak wind because of the static friction arisen from the inherent structure. In this case, the output performance is deteriorated as well as the generator cannot operate completely. In this work, a wind-driven triboelectric nanogenerator (wind-TENG) based on the fluttering of the PTFE strips is proposed to solve the aforementioned problems. At the minimum operating wind pressure of 0.05 MPa, this wind-driven TENG delivers the open-circuit voltage of 3.5 V, short-circuit current of 300 nA, and the associated output power density of 0.64 mW/m^2^ at the external load resistance of 5 MΩ. Such conditions can be used to light up seven LEDs. Moreover, this wind-TENG has been utilized as a direction sensor which can sense the direction at which the wind is applied. This work thus provides the potential application of the wind-TENG as both self-driven electronics and a self-powered sensor system for detecting the direction under environmental wind.

## 1. Introduction

The major concern pertaining to energy crises is that the worldwide demands on the natural resources which are used to power industrial society are increasingly growing [1,2]. As a consequence of the fuel shortage, energy harvesting technology, which is an efficient and sustainable technique to transform various environmental energies to useful electrical energy, has been developing [3]. Among the various renewable energy systems, wind energy has become one of the most efficient energy sources due to its abundance, ubiquity, and environmentally friendliness [4,5,6]. The conventional wind turbines, generally based on the mechanism of electromagnetic induction, convert kinetic energy of the wind into mechanical energy and then into electricity [7,8]. However, these kinds of energy harvesters have some limitations, such as heaviness, large volume, complicated structure, and low efficiency at low frequency, which cause these devices to be held back in small-scale applications under weak wind conditions [9]. For this reason, even though weak wind exists around us, it is easily discarded before harvest. For that reason, seeking a new type of energy harvester that can sensitively collect the small-scale wind is becoming increasingly necessary.

Recently, the triboelectric nanogenerator (TENG), first invented in 2012, has been utilized to harvest mechanical energy from our living environment into electricity based on triboelectrification and electrostatic induction [10,11,12,13,14,15,16,17,18,19], possessing the properties such as simple and low-cost fabrication process, high output performance, high efficiency at low frequency, and eco-friendly characteristic [20,21,22,23]. At this rate, harvesting wind energy to develop TENG technology is necessary because energy harvesting from wind can not only reduce the fabrication cost but also extend the potential applications of TENGs.

Transforming wind energy into electricity is regarded as one of the most popular research processes in TENGs. The first reported wind-driven TENG consists of two Al plates and fluorinated ethylene propylene (FEP) film which is fixed at one end and flutter to harness wind energy from the environment [24]. There have also been many reported TENGs which are based on the wind-driven effect [25,26,27,28,29,30,31,32]. Nevertheless, almost all of the reported devices are based on a bulk fluttering material structure. The dielectric material cannot properly flutter under the weak wind condition which reduces the efficiency and total virtue of the device at ambient situations.

In this work, we propose a wind-driven triboelectric nanogenerator based on the fluttering of the polytetrafluoroethylene (PTFE) strips dielectric layer. The wind-TENG consists of two Al electrode plates and the PTFE film as the dielectric layer with one end fixed and the other end left free-standing. To flap the fluttering layer of the device, wind is injected into the device through the conventional air gun. To enhance the fluttering rate and operational output under the weak wind, PTFE film is cut and snipped into strips at the free-standing end. The optimum length and width of the PTFE strips have been investigated to enhance the better output performance of the wind-TENG under the relevant wind pressure. The minimum operating wind pressure at which the device can harness the functional output has also been examined. The open-circuit voltage and short circuit current at the minimum operating wind pressure of 0.05 MPa are 3.5 V and 300 nA. The maximum output power density is 0.64 mW/m^2^ at the match resistance of 5 MΩ which can be used to illuminate seven commercial light-emitting diodes (LEDs). The direction can be sensed by injecting the wind into the integrated device, showing that the wind-TENG has potential in self-driven devices and self-powered sensor systems.

## 2. Materials and Methods

### 2.1. Fabrication of the Wind-TENG

The fabricated wind-TENG is based on the wind-driven fluttering PTFE film, two aluminum electrode plates and neodymium magnets as the spacer. The size of the Al plate is 60 mm × 30 mm with a thickness of 0.4 mm and that of neodymium magnet is 5 × 5 mm with a thickness of 0.5 mm. The water-assisted oxidation (WAO) process was conducted to grow the nanograss-like structure on the surface of the Al electrodes. Deionized water was put in a beaker and two Al electrodes were also added and then the beaker was heated to the temperature of 80 °C. The temperature was maintained at the same point for 1 h. The oxidized Al electrodes were then removed from the boiling water and completely dried them with nitrogen gas. The magnets were attached to the two corners of the aluminum electrode by using the fast-drying glue, at these corners, the PTFE film is fixed between the spacer magnets. The nitrogen gas was applied to the device at the wind pressure of 0.05 MPa. To flutter the PTFE film effectively under the weak wind, the free-standing end of the PTFE film was cut into strips and the length and width of the strips were adjusted to attain their optimum values.

### 2.2. Electrical Measurements

For the measurement of wind-TENG, wind was injected using a commercial air gun and the speed of the wind was measured via an anemometer. The open-circuit voltage and short-circuit current of the wind-TENG device were measured by using Keithley 6514 System electrometer (Cleveland, OH, USA).

## 3. Results and Discussion

**Figure 1:**
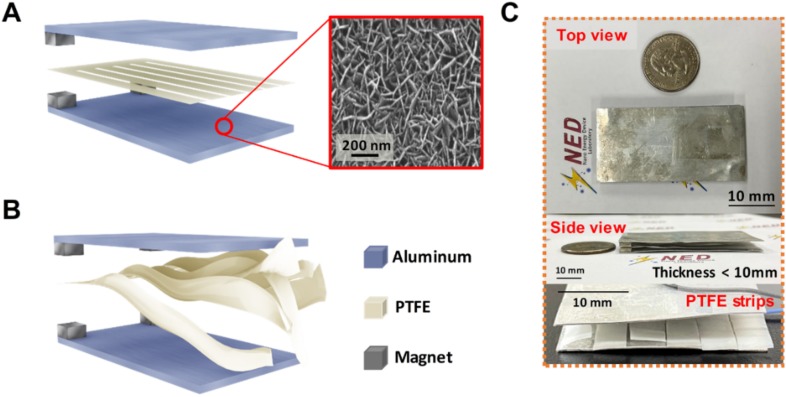
(**A**) Schematic diagram of the wind-TENG with SEM image showing the surface of Al electrode treated by WAO process. (**B**) Wind-TENG with the PTFE strips fluttering when the wind in injected. (**C**) The optical images of the wind-TENG taken from top view, side view and displaying the structure PTFE strips.

The schematic structure of the wind-TENG is illustrated in Figure 1A. The wind-TENG includes two aluminum electrodes with a PTFE film sandwiched between them, fixing one side of the PTFE film by using magnets. The Al plate played dual roles not only as a triboelectric surface but also as an electrode. A WAO process was introduced to the Al electrodes for the enhancement of output performance by increasing the effective contact area between PTFE strip and the Al electrodes. In the WAO process, two Al electrodes belonged to the wind-TENG were oxidized in deionized water at the temperature of 80 °C for about 1 h. The nanograss-like structures were formed on the surfaces of Al electrodes after the oxidization process as illustrated in the inset of Figure 1A. The electrical outputs of the wind-TENG comprising the bare Al and PTFE were presented in Supplementary Figure S1 for comparison and also added the SEM image of the bare Al electrode without WAO process. The magnets created the moving space for vibrating the PTFE film so that the free-standing end of the film can flap in the wind. This strip-like PTFE film can be vibrated with a high frequency and increase an effective contact area due to its cutting structure at the flapping end. Figure 1B showed the PTFE strips that were energetically fluttering even when the very weak wind was applied to the device, in which an air gun was utilized as a wind source with tunable wind flow. Figure 1C exhibits the top view, side view, and cut PTFE strips of the fabricated TENG. The dimension of the wind-TENG is 60 × 30 mm, with the thickness of merely 6 mm, and the detailed measurements are mentioned in the experimental section.

**Figure 2:**
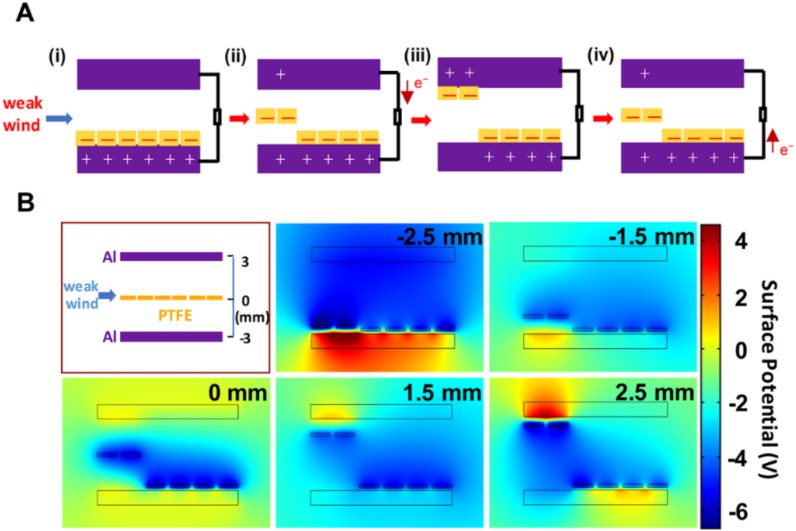
(**A**) Working mechanism of the wind-TENG (**B**) Finite-element simulation of the surface potential variation for wind-TENG.

The working mechanism of the fabricated wind-TENG was illustrated in Figure 2A. At the initial state, the surfaces of both bottom Al electrode and PTFE strips completely contacted with each other, transferring electrons from Al to PTFE strips and the positive and negative triboelectric charges were appeared on the Al and PTFE surfaces, respectively, according to the triboelectric series [33,34]. No electron flow occurred in the external circuit when the produced triboelectric charges with opposite polarities are fully balanced as shown in Figure 2A(i). As the wind was applied to the device, the separation between the bottom electrode and PTFE strips starts and causes the potential difference between two electrodes. This potential difference drove the electrons to flow from the top electrode to bottom electrode through the external load (Figure 2A(ii)) until the PTFE strips completely reach the top electrode (Figure 2A(iii)). When the PTFE strips move back from the top electrode to the bottom electrode, the reversed electrons flowed to its original state (Figure 2A(iv)). Through this series of vibrating processes, the wind-TENG can produce the alternating current (AC) in the external circuit as the electrons flow back and forth between two Al electrodes. In the mentioned working mechanism, to clear the operating procedure of the flapping film under the weak wind, only two PTFE strips fluttered up and down between the two electrodes while other strips still remained at the bottom electrode. The other strips were unable to escape from their existing position due to the electrostatic attraction upon the film. The working mechanism of the wind-TENG with the bulk PTFE is also expressed in Supplementary Figure S2. The bulk film was hard to move up under the weak wind and it can solely generate extremely low output. To verify the working principle of the wind-TENG, the finite element method was employed to carry out the theoretical simulation in an air condition through COMSOL Multiphysics software. Figure 2B depicts a simplified schematic and five computed results showing the surface charge distribution and electric potential variation between two electrodes of TENG at different positions of the moving PTFE strips at −2.5, −1.5, 0, 1.5, and 2.5 mm, respectively. These simulation results were in accord with the charge generation and distribution described in the working mechanism in Figure 2A.

**Figure 3:**
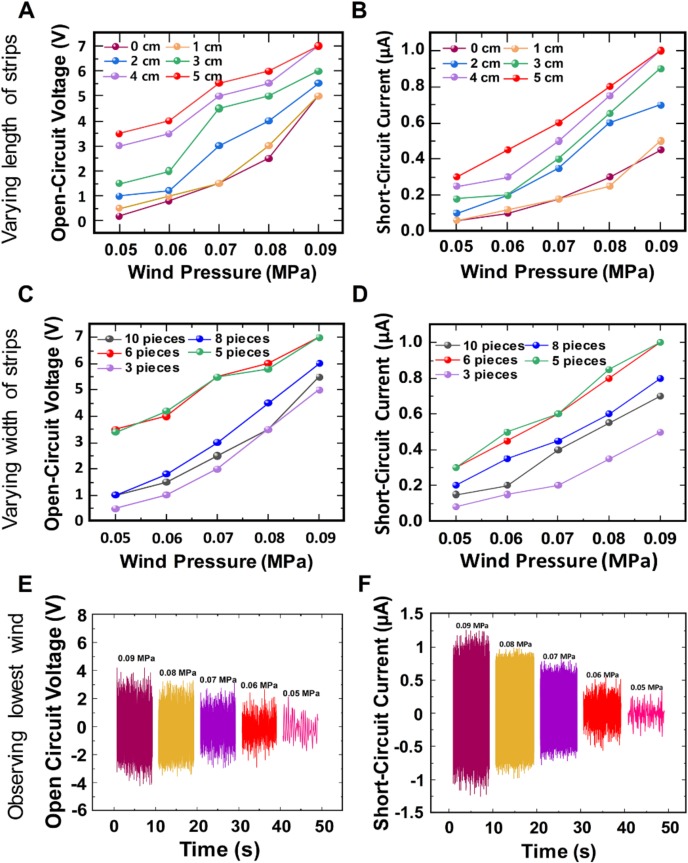
Examining the optimum length of the PTFE strips under different weak winds showing (**A**) Open-circuit voltage and (**B**) Short-circuit current; Examining the optimum width of the PTFE strips under different weak winds showing (**C**) Open-circuit voltage and (**D**) Short-circuit current (**E**) Open-circuit voltage and (**F**) Short-circuit current for observing the minimum wind pressure.

The output dependence of the wind-TENG device on varying the length and width of PTFE strips was observed by conducting following experiments. First, the length of the PTFE film was examined by cutting into different values to compare with the output performance of the same PTFE film with no cutting under weak wind. Since the length of the PTFE film is 6 cm, each film was snipped into different dimensions of 1 cm, 2 cm, 3 cm, 4 cm, and 5 cm which resemble 15%, 30%, 50%, 65%, and 80% of the full-length of PTFE film respectively. The width of each PTFE strip was 0.5 cm which splits one end of the film into 6 pieces. The images displaying different length of the cut PTFE film are shown in Supplementary Figure S3A. Figure 3A,B shows the line graphs describing the open-circuit voltages and short-circuit currents of the wind-TENG with the varying length of the strips under different wind pressures at the direction of lateral side. The nitrogen gas was injected to the wind-TENG with different pressures of 0.05 MPa, 0.06 MPa, 0.07 MPa, 0.08 MPa, and 0.09 MPa which can be converted to speeds of 1.5 m/s, 1.8 m/s, 2.1 m/s, 2.4 m/s, and 2.7 m/s respectively, using an anemometer. These wind speeds were regarded as those under the average wind speed in our environment [35]. To show the advantage of this device structure, the output performances of the wind-TENG were observed at the wind speeds under the natural wind in our environment. As shown in Figure 3A, the open-circuit voltages of the wind-TENG with 5 cm-length of the PTFE strips showed the highest values which were measured within the various weak wind conditions. It is obvious that the wind-TENG operating with the PTFE strips can produce 20–70% larger outputs than the device with the bulk film from the 0-cm condition. This result was attributed to the size and weight of the PTFE strips fluttering with the weak wind. From the trailing edge of the short film, only single-contact was induced by the same input, while the longer fluttering film created the double-contact behavior in the middle and at the end [26]. The same tendency occurred for the short-circuit current as shown in Figure 3B due to the fact that the electrical outputs were affected by the change of effective contact area. The optimum width of the PTFE strips that can generate better output was also investigated. The length of the PTFE strips, 5 cm, was set constant as this length showed the best output in checking the optimum length of the strips. The width of the PTFE strip was altered into 0.3 cm (10% of the entire width of PTFE), 0.4 cm (13%), 0.5 cm (16%), 0.6 cm (20%), and 1 cm (30%) which attained 10 pieces, 8 pieces, 6 pieces, 5 pieces, and 3 pieces of PTFE strips at one end, respectively. The flutter speed of the strips partially depended on the mass ratio of the fluttering material [36]. The PTFE strips were unable to move if the weight was larger than the applied wind. Thus, the dielectric material including 3 pieces of PTFE strips showed the least flutter speed and generates lower output comparing with other width cases. On the contrary, the flutter speeds of the PTFE films with 8 pieces and 10 pieces were faster for the reason of each strip possessing smaller mass ratio. However, the fluttering motion became random and chaotic with inducing irregular contact area and generated the lower output performance in overly lightweight cases. By observing the Figure 3C,D, the above discussions were proved and the width of the PTFE strips from 16% to 20% (5 pieces and 6 pieces) represented the enhanced outputs. The reported papers mostly described the output dependence of the wind device on increasing of the wind speed [37,38]. In contrast, this paper focused on the weak wind speed and found the lowest point of the wind at which the wind-TENG was able to operate regardless of the output values. As we can see in Figure 3E,F, the wind pressure with 0.05 MPa was perceptible by using this fabricated wind-TENG.

**Figure 4:**
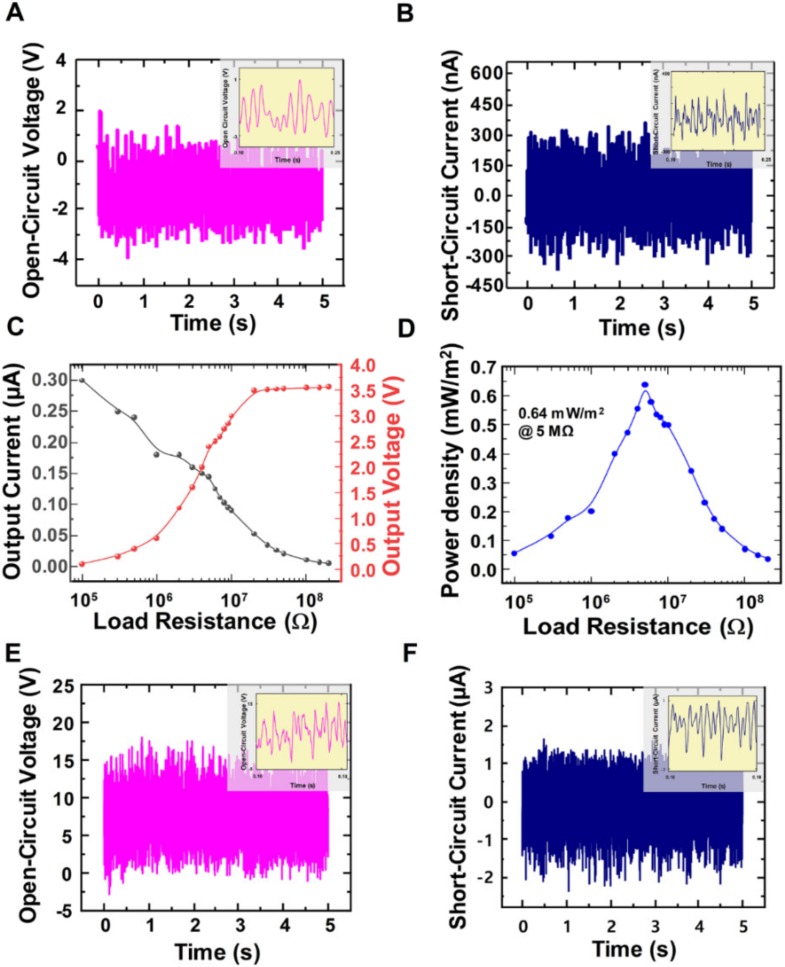
(**A**) Open-circuit voltage and (**B**) Short-circuit current at the minimum operating wind pressure of 0.05 MPa. (**C**) Load resistance dependency of the output voltage and current at wind pressure of 0.05 MPa. (**D**) Load resistance dependency of the output power at wind pressure of 0.05 MPa. (**E**) Open-circuit voltage and (**F**) Short-circuit current at the high wind pressure of 0.2 MPa.

The output performances of the wind-TENG with 5 cm-length and 0.5 cm-width of PTFE strips (6 pieces) were measured under the minimum operating wind pressure of 0.05 MPa. The open-circuit voltage and the short-circuit current represented the values of 3.5 V and 300 nA, (Figure 4A,B) respectively. To further examine the performance of the wind-TENG, the electrical outputs depending on the load resistance were also investigated within the range from 100 kΩ to 200 MΩ. The output voltage increased with the enhancing load resistance and remained almost constant at the higher resistance over 200 MΩ while the output current showed the reverse manner (Figure 4C). The voltage and current values were multiplied to extract the output power. The maximum output power density of 0.64 mW/m^2^ was obtained with an internal resistance of 5 MΩ with the intensity of a gentle wind. The output performances were also measured with this wind-TENG under the high wind velocity to prove that this fabricated device is comparable with other wind energy harvesting devices. Figure 4E,F present the open-circuit voltage of 17 V and short-circuit current of 2 µA from the wind-TENG at the wind pressure of 0.2 MPa with the wind speed of 6 m/s. The output performances related to the external load resistance were also expressed in Supplementary Figure S4A,B. The maximum output power density was 6.25 mW/m^2^ at the load resistance of 5 MΩ, which was identical to the internal resistance value from a gentle wind.

**Figure 5:**
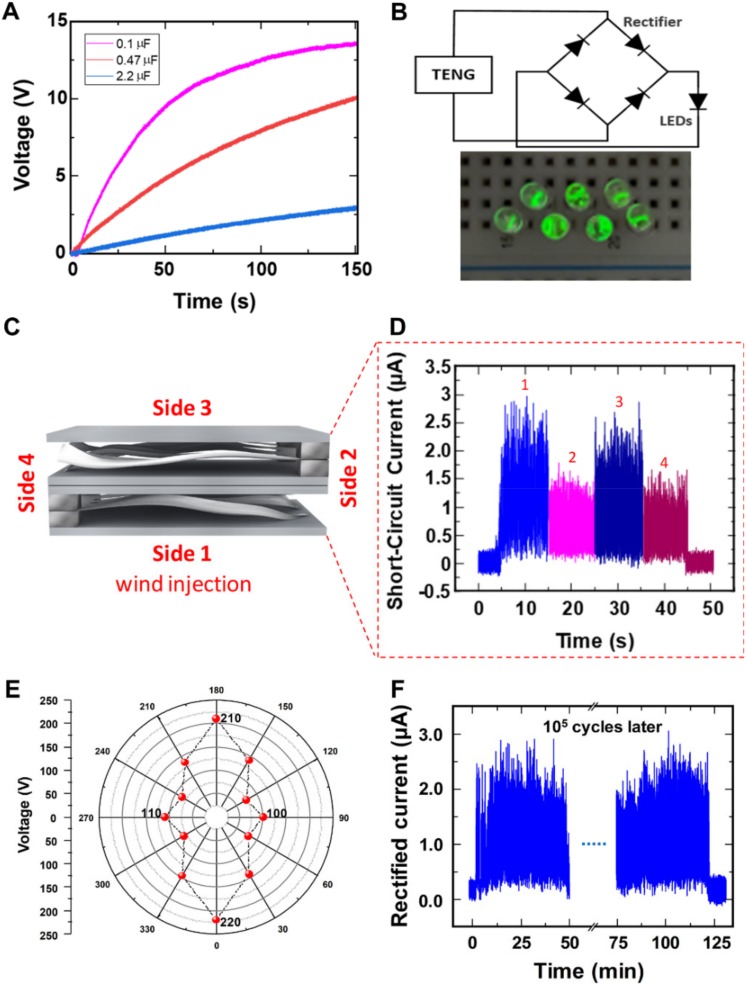
(**A**) Capacitor charging of the wind-TENG with various capacitances. (**B**) Circuit diagram and actual image for powering LEDs. (**C**) Schematic diagram of the integrated device consisting of two TENGs stacking. (**D**) Short-circuit current when the wind is injected into four individual sides of the integrated TENG. (**E**) Polar graph displaying the open-circuit voltage indicating the directionality of wind-TENG. (**F**) Durability test results for the wind-TENG.

The applicability of the wind-TENG was certified by the charging of the capacitors and the real-time operation of LED illumination, which were simple and efficient tasks to prove the reliability of the TENGs. The charging performance of the wind-TENG for different capacitors at the wind pressure of 0.05 MPa was investigated, as illustrated in Figure 5A. The output from the wind-TENG was rectified by utilizing a full-wave bridge rectifier circuit and applied to a capacitor. The various capacitors with capacitances of 0.1 µF, 0.47 µF, and 2.2 µF were charged in 150 s, respectively. The seven LEDs array was continuously turned on when the rectified circuit is connected to LEDs. To detect the direction from omnidirectional wind, the wind-TENG was integrated by utilizing two devices connected in parallel through each bridge rectifier. Two wind-TENGs were stacked in 180° rotation, with PI film sandwiched to prevent short-circuit between two devices as depicted in Figure 5C. The wind was injected to four individual sides of the integrated device to compare the generated outputs by changing the air-injecting direction, recording each side to side 1, 2, 3, and 4, respectively. According to the dimension difference of the device, different fluttering actions occurred to each side of the device under the same weak wind condition. As displayed in Figure 5D, the short-circuit currents of side 1 and 3 become merely double when compared to side 2 and 4. The same trend happened to the open-circuit voltages which were presented in Figure S5. As the device was shaped with the rectangular-form, the fluttering rates of side 1 and 3 were similar and same for side 2 and 4. The PTFE strips fluttered freely and generated more output when the wind was injected to side 1 and 3 while the strips from the fixed end at side 2 and 4 barely fluttered causing the lower output than the others. Figure 5E shows the polar graph including the voltage values obtained when the wind is injected from various directions. The outputs display the higher values when the wind inlet and the direction of the wind are concurrent and the values eventually decrease as long as the wind inlet and the direction of the applied wind are not at the right angle, in this way, the device can be utilized as the direction sensor which detects the wind applied from all directions. A durability test at the wind pressure of 0.05 MPa manifests the stability and reliability of the wind-TENG device (Figure 5F). The rectified current values do not show any observable deterioration even after 10^5^ cycles of oscillation.

## 4. Conclusions

In summary, this work presented a wind-driven triboelectric nanogenerator based on the fluttering of PTFE strips to harvest the mechanical energy from notably weak wind speed comparable to the intensity of a gentle wind. By observing the optimum length and width of the PTFE strips, the wind-TENG can operate at the minimum wind pressure of 0.05 MPa with the wind speed of 1.5 m/s. The open-circuit voltage and short-circuit current of the wind-TENG at 1.5 m/s were shown with the values of 3.5 V and 300 nA, respectively and the maximum output power density of 0.64 mW/m^2^ was represented under the load resistance of 5 MΩ. The fabricated wind-TENG can be used to charge various capacitors and light up seven commercial LEDs. The output performances at the high wind speed which value was over the average natural wind speed, were also measured to prove the device can generate higher output and be comparable with other wind-driven devices. Moreover, by integrating the wind-TENG in a stacking structure through a parallel connection, the directionality of omnidirectional wind was favorably detected. On account of the above merits, the wind-TENG can be applied in our environment to scavenge even a weak wind energy which is converted into electricity for powering the electronics or charging energy storage devices. This work illustrated the high potential in the practical applications of TENG for extremely weak wind energy harvesting.

## Supplementary Materials

The following are available online at https://www.mdpi.com/2072-666X/11/4/414/s1, Figure S1: (A) Schematic diagram of the wind-TENG with SEM image showing the surface of bare Al electrode without WAO process. (B) Open-Circuit voltage and (C) Short-Circuit current of wind-TENG comprising bare Al and PTFE at the minimum operating wind pressure of 0.05 MPa, Figure S2: Working mechanism of the wind-TENG with the bulk PTFE under weak wind, Figure S3: Optical images of PTFE films displaying, Figure S4: (A) Load resistance dependency of the output voltage and current at wind pressure of 0.2 MPa. (B) Load resistance dependency of the output power at wind pressure of 0.2 MPa, Figure S5: Open-circuit voltages when the wind is injected into (A) Side 1 (B) Side 2 (C) Side 3 and (D) Side 4 of the integrated TENG.
