# Supplementary material for: Omnidirectional Triboelectric Nanogenerator Operated by Weak Wind towards a Self-Powered Anemoscope
Source: Micromachines (Basel). 2020 Apr 14;11(4):414. doi: 10.3390/mi11040414 (PMC7231358; doi:10.3390/mi11040414)
Supplement: Supplementary file 1 [file micromachines-11-00414-s001.pdf]

micromachines

Supplementary Materials: Omnidirectional Triboelectric Nanogenerator Operated by Weak Wind Towards a Self-Powered Anemoscope

Nay Yee Win Zaw [1], Hyeonhee Roh [1], Inkyum Kim [1], Tae Sik Goh [2,*] and Daewon Kim [1,*]

[1] Department of Electronic Engineering, Institute for Wearable Convergence Electronics, Kyung Hee University, Yongin 17104, Korea

[2] Department of Orthopaedic Surgery and Biomedical Research Institute, Pusan National University Hospital, Busan 49241, Korea

* Correspondence: daewon@khu.ac.kr (D.K.); taesikgoh@gmail.com (T.S.G.)

Received: 29 February 2020; Accepted: 11 April 2020; Published: date

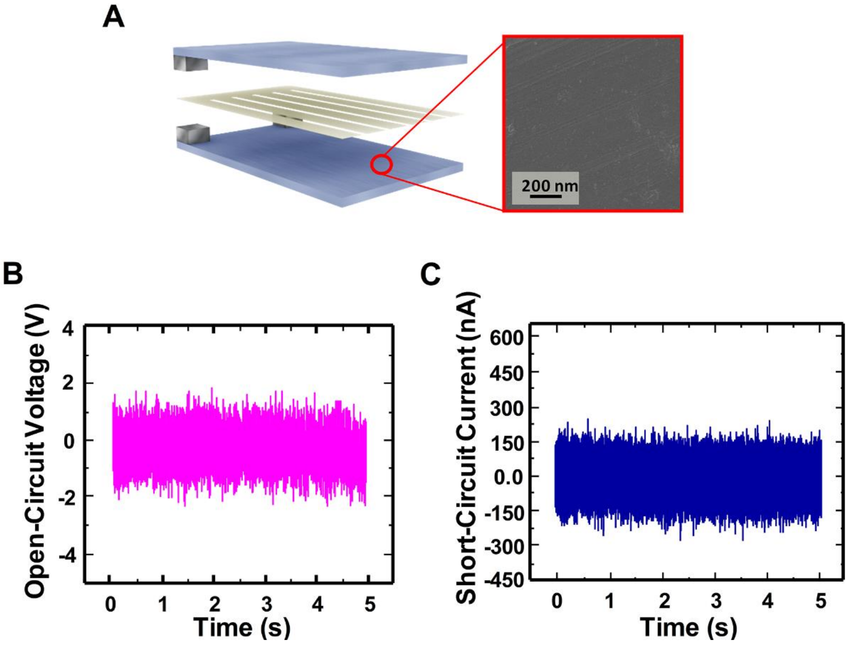

Figure S1. (**A**) Schematic diagram of the wind-TENG with SEM image showing the surface of bare Al electrode without WAO process. (**B**) Open-Circuit voltage and (C) Short-Circuit current of wind-TENG comprising bare Al and PTFE at the minimum operating wind pressure of 0.05 MPa.

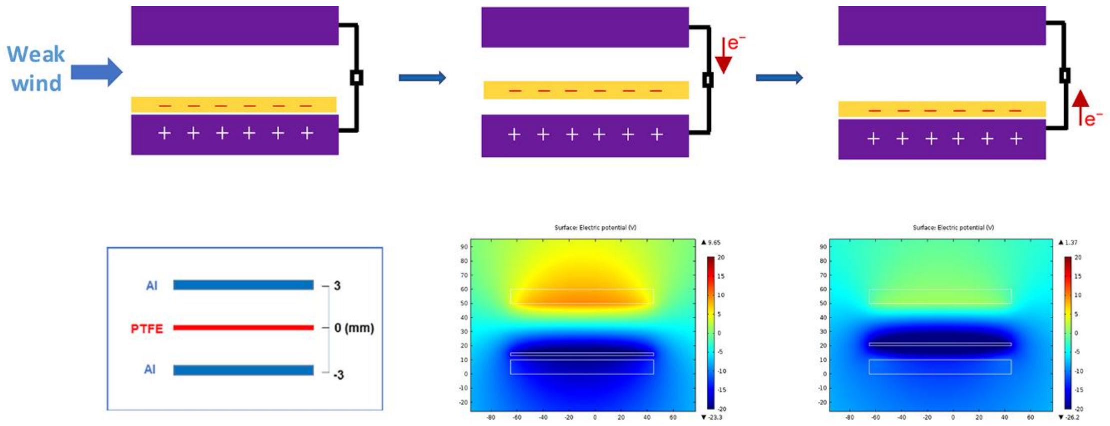

Figure S2. Working mechanism of the wind-TENG with the bulk PTFE under weak wind.

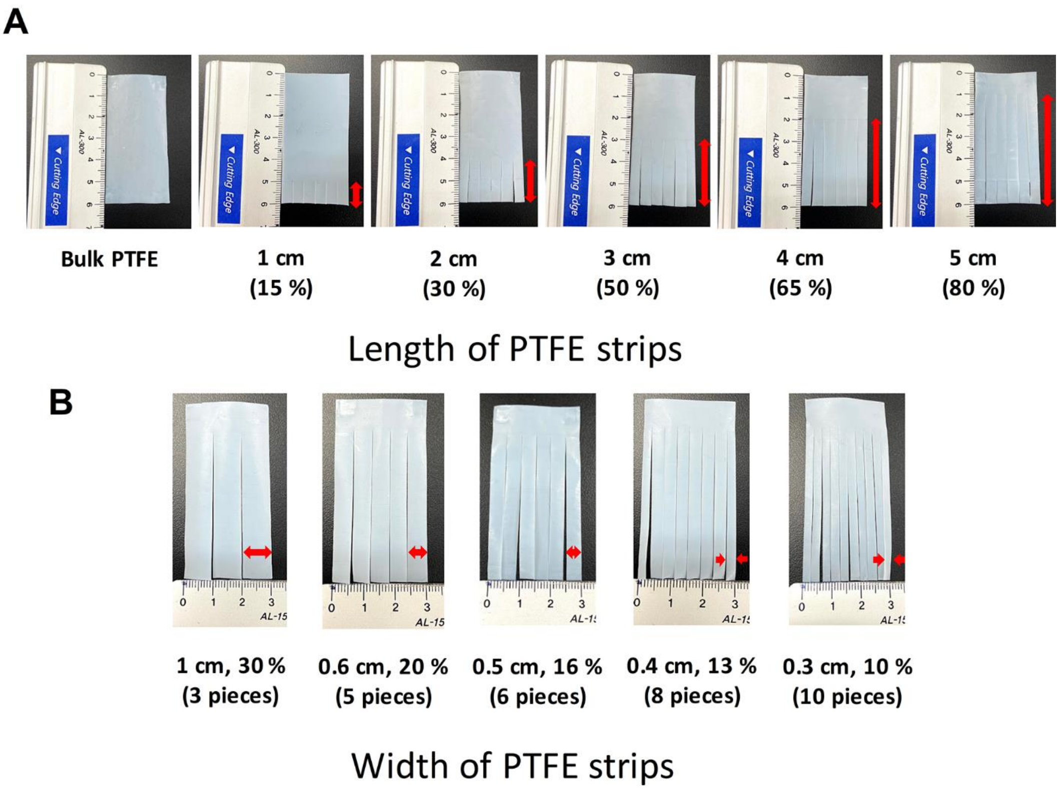

Figure S3. Optical images of PTFE films displaying (**A**) Length of PTFE strips and (**B**) Width of PTFE strips.

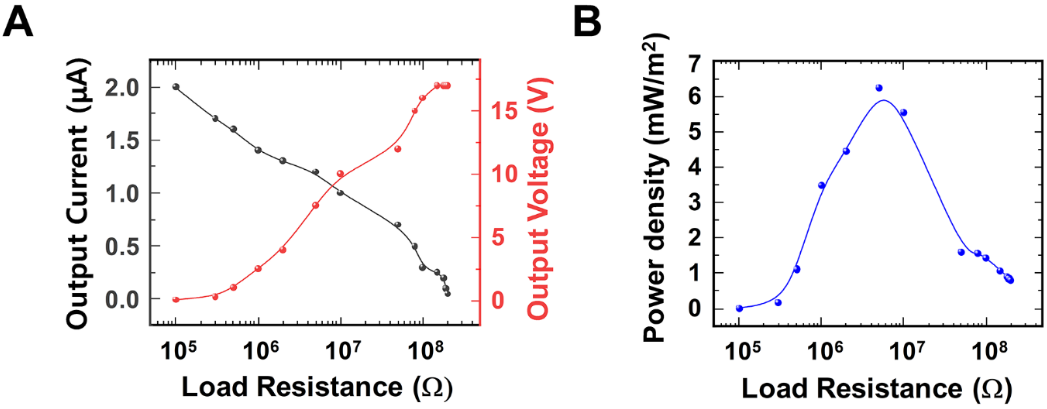

Figure S4. (**A**) Load resistance dependency of the output voltage and current at wind pressure of 0.2 MPa. (**B**) Load resistance dependency of the output power at wind pressure of 0.2 MPa.

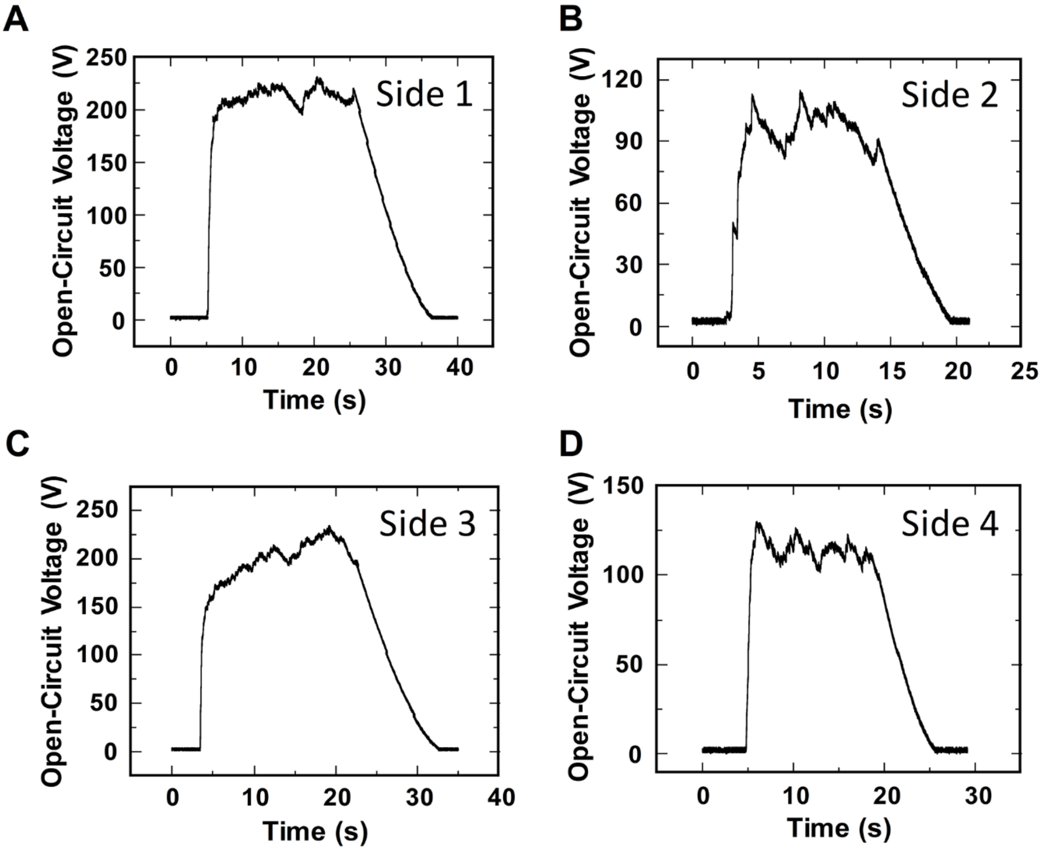

Figure S5. Open-circuit voltages when the wind is injected into (**A**) Side 1 (**B**) Side 2 (**C**) Side 3 and (**D**) Side 4 of the integrated TENG.
